# Slow but Steady: Similar Sit-to-Stand Balance at Seat-Off in Older vs. Younger Adults

**DOI:** 10.3389/fspor.2020.548174

**Published:** 2020-10-26

**Authors:** Lizeth H. Sloot, Matthew Millard, Christian Werner, Katja Mombaur

**Affiliations:** ^1^Optimization, Robotics & Biomechanics, Institute of Computer Engineering, Heidelberg University, Heidelberg, Germany; ^2^Center for Geriatric Medicine, Agaplesion Bethanien Hospital Heidelberg, Heidelberg University, Heidelberg, Germany; ^3^Department of Systems Design Engineering, Department of Mechanical and Mechatronics Engineering, University of Waterloo, Waterloo, ON, Canada

**Keywords:** sit-to-stand, balance, static, dynamic, foot placement estimator, coordination, elderly, stability

## Abstract

Many older adults suffer injuries due to falls as the ability to safely move between sitting and standing degrades. Unfortunately, while existing measures describe sit-to-stand (STS) performance, they do not directly measure the conditions for balance. To gain insight into the effect of age on STS balance, we analyzed how far 8 older and 10 young adults strayed from a state of static balance and how well each group maintained dynamic balance. Static balance was evaluated using the position of the center-of-mass (COM) and center-of-pressure (COP), relative to the functional base-of-support (BOS). As the name suggests, static balance applies when the linear and angular velocity of the body is small in magnitude, in the range of that observed during still standing. Dynamic balance control was evaluated using a model-based balance metric, the foot-placement-estimator (FPE), relative to the COP and BOS. We found that the older adults stay closer to being statically balanced than the younger participants. The dynamic balance metrics show that both groups keep the FPE safely within the BOS, though the older adults maintain a larger dynamic balance margin. Both groups exhibit similar levels of variability in these metrics. Thus, the conservative STS performance in older adults is likely to compensate for reduced physical ability or reduced confidence, as their dynamic balance control does not seem affected. The presented analysis of both static and dynamic balance allows us to distinguish between STS performance and balance, and as such can contribute to the identification of those older adults prone to falling, thus ultimately reducing the number of falls during STS transfers.

## 1. Introduction

The ability to get up from a chair is a fundamental prerequisite to perform daily activities and function independently. Unfortunately, difficulty moving from a sit to a stand (STS) is common among older adults, and affects the lives of over 6% of those living independently as well as over 60% of long-term care residents (Jeyasurya et al., 2013). Poor STS performance can make ambulatory older adults prisoners in their chairs and can result in falling. More specifically, more falls occur during STS (including stand-to-sit) compared to walking, especially in residents with higher fall frequency (Rapp et al., 2012; Pozaic et al., 2016; van Schooten et al., 2017). As falls are the number one cause of injuries in older adults over the age of 65, it is imperative to understand and improve their STS performance (Janssen et al., 2002; Millor et al., 2014).

STS is considered the most mechanically demanding task of common daily activities, requiring leg muscle strength, coordination, and balance control (Riley et al., 1997; Millor et al., 2014). Limitations in any one of these factors is suggested to cause poor STS ability, resulting in STS attempts that fail, leaving older adults to sit back down or take a step if possible, potentially creating an even more unstable situation. Surprisingly, metrics to analyze balance during STS have yet to be found to identify those at risk of falling.

Clinical tests commonly measure the duration of, or ability to perform, a number of STS movements, which yields little information regarding any underlying problems (Bohannon, 2012; Silva et al., 2014). Biomechanical studies have described the STS motion, different STS compensatory strategies, and evaluated the effect of factors, such as chair height and foot placement on STS difficulty (Aissaoui and Dansereau, 1999; Janssen et al., 2002; Millor et al., 2014; Boukadida et al., 2015). Only a few studies have aimed to evaluate balance during STS using metrics such as transfer duration, body or trunk dynamics around seat-off, or the location of the body's center of mass or pressure relative to the ankle at seat-off (Moxley Scarborough et al., 1999; Åberg et al., 2010; Akram and McIlroy, 2011; Fujimoto and Chou, 2014). However, these metrics are unable to discern the difference between movement and balance. In addition, most of these studies measure STS with the arms crossed at the chest, while this might affect balance and does not reflect STS movement in daily life.

Recently, a model-based balance metric called the foot placement estimator (FPE), originally developed to study balance in bipedal robotics, was applied to assess dynamic balance during walking in healthy adults, children and patients with movement disorders (Wight et al., 2007; Millard et al., 2009, 2012; Bruijn et al., 2013). The 3D FPE (Millard et al., 2012) uses a 3D inverted pendulum model to calculate where the center-of-pressure (COP) should be placed with respect to the center-of-mass (COM) so that the participant can passively reach a statically balanced standing position (Millard et al., 2012). Although the FPE is similar to the capture-point (Pratt et al., 2006) and the extrapolated center of mass (Hof, 2008), it improves upon these methods by taking both linear and angular momentum into consideration. The FPE has been validated for analyzing gait but the metric has not yet been applied to quantify dynamic balance during STS. The FPE can express the dynamic balance margin relative to the base-of-support (BOS), taking into account specific foot placement and dynamics of the STS movement, to predict how close individuals come to taking a corrective step. In addition, the relative placement of the COP to the FPE can be used to evaluate how well individuals control their direction of travel and speed.

The aim of this paper is to compare how accurately older adults control their balance during STS compared to young adults, by analyzing the motion of the COM, COP, and FPE relative to the BOS of individuals. STS transfers were performed with both more natural arm position (hanging at the side) and arms crossed at the chest to allow for comparison to literature. First, we evaluated static balance by calculating how far each participant was from meeting the conditions for static balance at seat-off, by calculating how far the COM ground projection (COM_GP_) is from the BOS, the distance between the COM_GP_ and the COP, the COM speed, and finally the average angular speed of the body. Second, we used the FPE to evaluate how well dynamic balance was controlled by evaluating the distance between FPE and BOS as well as how accurately participants tracked the FPE with their COP. We expect that older adults who struggle with STS motions will stay closer to being statically balanced but will struggle to remain dynamically balanced and thus will come closer to taking a compensatory step than younger adults. We also expect older adults to display more variability in all static and dynamic balance measures.

## 2. Methods

Sit-to-stand transfers were performed in two different conditions: with arms relaxed at the side (Side) as a more natural, primary condition and with arms crossed at the chest (Chest) for comparison to other STS studies in literature. For the Side condition, participants were instructed to start each STS with arms at their side, but no further instructions were given on arm movement during the task. Participants were asked to perform five consecutive STS movements at their own pace, starting and stopping each cycle with about 2 s of sitting and standing still. Still standing trials were also performed so that the bounds on the various static balance metrics, described in section 2.3, could be established.

A stool (used for marker visibility reasons) was placed at one force plate. As seat height is known to affect STS difficulty (Janssen et al., 2002), it was set to the height of the knee epicondyles, which was similar between groups (Y: 49 ± 4 cm, E: 50 ± 4 cm). Participants were instructed to position their feet at their preferred position on the second force place and to not move their feet during the entire experiment. It is important to note that the foot placement relative to the stool, which is known to affect STS difficulty, was similar between young and older adults (Supplementary Figure 1) (Janssen et al., 2002).

Data were simultaneously collected using the two ground-embedded force plates at 900 Hz (Bertec, Columbus, OH, USA) and a passive motion capture system at 150 Hz (type 5+ cameras, Qualisys, Gothenburg, Sweden). Motion capture markers (14 mm) were placed on each participant according to the IOR full body model (Cappozzo et al., 1995; Leardini et al., 2011), with extra iliac crest and greater trochanter markers to guarantee tracking throughout the STS motion. Marker and force data were filtered with a bi-directional low-pass Butterworth filter (with a cut-off frequency of 10 Hz). The body COM position, COM velocity, moment-of-inertia about the COM, and angular momentum about the COM was calculated using the IOR full body human model, with a separate upper and lower trunk, in Visual3D (v6, C-motion, Inc., Germantown, MD, USA). As most arm markers were covered up during the Chest condition, the weight of the arm segments was added to the upper trunk in this condition.

To characterize the older participants, we administered established clinical tests and questionnaires. First we determined the participant's frailty level according to the Clinical Frailty Scale (Rockwood et al., 2005) and cognitive function using the Mini-Mental State Examination (Folstein et al., 1975; Tombaugh and McIntyre, 1992) as part of the screening process. After the STS evaluation and a break, participants performed the short physical performance battery (SPPB) (Guralnik et al., 1994) to assess functional ability. At the end of the session, we administered the Barthel Index of activities of daily living (Mahoney and Barthel, 1965) as another measure of functional ability and evaluated fear of falling using the falls efficacy scale international (FES-I) (Hauer et al., 2010) and asked for the number of falls in the last year (see Table 1).

**Table 1:** Older adult (O) characteristics.

	**Age**	**Frailty**	**Cognition**	**Functional ability**			**Falling**	
	**(years)**	**CFS**	**MMSE**	**Barthel I**	**SPPB**	**Aid**	**falls/years**	**FES-I**
		**(impair:+)**	**(impair:-)**	**(impair:-)**	**(impair:-)**			**(afraid:-)**
O1	80–85	3	29	85	12	Cane	1	16
O2	90–95	5	29	95	10	None	1	11
O3	75–80	2	29	100	10	None	0	11
O5	80–85	3	27	100	7	Stick^*^	1	13
O6	65–70	3	24	95	8	Cane	0	17
O7	80–85	4	21	95	8	Rollator	0	12
O8	65–70	1	29	100	11	None	0	9
O9	75–80	3	29	100	12	Cane^*^	0	9

With the age range indicated for anonymity; CFS the Clinical Frailty Scale testing the frailty level with scores 1–9 (Rockwood et al., 2005); MMSE the Mini-Mental State Examination testing the cognitive function with scores 0–30 (Folstein et al., 1975; Tombaugh and McIntyre, 1992); Barthel I the Barthel Index of activities of daily living with scores 0–100 (Mahoney and Barthel, 1965); SPPB the short physical performance battery with scores 0–12 (Guralnik et al., 1994); Assistive device (aid) used in daily life (note: most of these devices were only used for longer distances outside the house, no assistive devices were used in the study and * these assistive devices are only used occasionally; number of falls in the last year; FES-I the falls efficacy scale international, evaluating the fear of falling with scores 7–28 (Hauer et al., 2010). For each scale it is indicated if more impaired is a higher or a lower value. For brevity a Nordic walking stick is referred to as a “stick”.

### 2.1. Participants

Ten able-bodied younger adults (28 ± 5 years, 4 females, 24 ± 2 kg/m^2^ BMI) and eight older adults (79 ± 8 years, 6 females, 29 ± 6 kg/m^2^ BMI) were included (see Table 1). Younger participants had to be between 18 and 45 years of age and older participants above 65 years. Participants were excluded from either group if they had neurological, cardiovascular, metabolic, visual, auditory, mental or psychiatric impairments or injuries that made the participant unable to independently perform the sit-to-stand task or walk short distances in the lab. Older participants were excluded if their frailty level was more than moderately frail (Clinical Frailty Scale score ≥6) (Rockwood et al., 2005) and if they were severely cognitively impaired (Mini-Mental State Examination ≤17) (Folstein et al., 1975; Tombaugh and McIntyre, 1992). It should be noted that participant O6 presented with chronic hemiplegia, but was included as the participant was able to independently perform STS. Since stroke is the leading cause of long-term disability in older adults, the inclusion of O6 contributed to creating a more representative sample of older adults. The protocol was approved by the Institutional Review Board of the medical faculty of Heidelberg University and all participant provided written informed consent.

### 2.2. Movement Segmentation

Most of our analysis focuses at the time of seat-off which is also the time associated with the fastest movements during STS. As such our analysis is sensitive to how accurately and consistently the time of seat-off is identified. Given the considerable variability among participant's movements, we developed a two-stage algorithm to automatically identify STS transfers and to consistently identify the times of initiation, seat-off, and standing.

In the first stage of the algorithm we used the k-means++ algorithm (Arthur and Vassilvitskii, 2007) to identify candidate motion sequences by clustering both the COM height and the vertical force recorded by the stool's force plate into three clusters. The three COM height clusters were identified and labeled using the mean value of each cluster: the cluster with the lowest mean height is the seated-height cluster, the cluster with the highest mean height is the standing-height cluster, while everything else is in the crouching-height cluster. Similarly the three force plate clusters were identified and labeled using the mean values of each cluster: the cluster with the largest vertical force reading is the seated-force cluster, the cluster with the lowest vertical force reading is the standing-force cluster, and everything else is in the transition-force cluster.

This process yields two vectors in which every point in time has two labels: one label from the COM height clusters, and another from the force plate clusters. Candidate motion sequences were identified by finding all time sets with standing-height label (at least 0.5 s in length) that are connected backwards in time to a seated-force label (also at least 0.5 s in length) with only a single transition from the transition-force cluster to the standing-force cluster in between. Sequences which met this criteria but included times at which the feet broke contact with the ground (at least 3 of the 6 foot markers must move by <15 mm in the vertical direction to be acceptable) were rejected.

In the second stage the event times of the motion sequences were refined. The beginning of the STS transfer was identified as the point in time at which the COM speed was 1 cm/s higher than the minimum value observed in the sitting-force set. The time of seat-off is defined by the point in time in which the vertical force of the stool force plate is within 1 N of the value it registers during the standing-height cluster (when the participant is not in contact with the stool). Stance is defined by the point in which the participant's COM is within 1 cm of the median height of the standing cluster and moving upwards with a speed of <1 cm/s.

This combination of clustering and adaptive thresholding allowed us to consistently identify movement segments from even the most variable of our participants while discarding as little data as possible. In the Side condition we had to reject 1 trial from O1 (sit-back), 1 trial from O5 (short still sitting), and 7 trials from O6 who presented with hemiplegia (1 with short still standing and 6 with a compensatory step). Since the difficulties O6 faced were clear during the data collection we measured additional trials until 5 successful STS transfers were recorded. In the Chest condition we had to reject 2 trials from O6 (1 with short still sitting and 1 sit-back) and 1 trial from O7 (short still sitting). While our analysis focused on the moment of seat-off, data on the transfer between seat-off to stance, and the median stance values are presented in the figures for context.

### 2.3. Balance Analysis

The definition of what constitutes balanced movement is task dependent: when a gymnast performs a tumbling routine the pelvis may contact the ground during balanced movement, in contrast, during STS transfer from a chair this would indicate that a fall has occurred. To analyze STS transfers we will adopt the following definitions:

**Definition:** The participant has *fallen* if any part of their body other than the bottoms of their feet touch the ground.

**Definition:** The participant is *statically balanced* if they have not fallen, and the linear and angular speed of their body is small. Here the term ‘small' is taken to mean within the bounds observed during still standing.

**Definition:** The participant is *dynamically balanced* if they are not statically balanced but eventually become statically balanced without falling.

An STS transfer contains a mixture of static and dynamic balance: the movement begins and ends in a state of static balance and between participants may be dynamically balanced. Accordingly we have defined measures to let us determine if a participant is statically balanced, and if not, to analyze their dynamic balance. The static balance measures quantify the conditions that must be met for an object to be in a state of static balance. The conditions necessary to analyze dynamic balance cannot be measured directly but can be interpreted using a model. As STS transfers include appreciable amounts of both linear and angular momentum, we make use of the FPE (Millard et al., 2012) because this model takes both of these components into account. The following text will briefly cover mathematical details of the static and dynamic balance metrics used in this analysis. For further details see (Wight et al., 2007; Millard et al., 2009, 2012).

We will use a short-hand notation to refer to the various points of interest: the COM is represented by point C, the COM_GP_ by point G, the COP by point P, the point of BOS polygon that is nearest to a hypothetical point A is B(A). A position vector (*r*) from point G to P is given by _G_*r*_P_ (_from_*r*_to_) while a position vector from the inertial frame to the COM is given by *r*_C_ (the inertial frame is omitted from the subscript labeling). Velocity vectors follow the same subscript convention with *v* used for linear velocity and ω used for angular velocity. A signed scalar is given by *d* while strictly positive scalars (such as speeds and distances) are indicated by applying the Euclidean norm operator (||·||_2_) to the vector of interest.

To see if a balance metric has values that are consistent with static balance we compared the results to the range spanned during still standing. To this end, we analyzed 10.0 s of quiet standing data in a sub-set of the participants (Y1, Y2, Y6, O1, O2, O6, O7, O9) and calculated the maximum and minimum values of all metrics, averaged across subjects, observed during the still standing trials. For signed metrics we use the larger of the two bounds to result in a symmetric region.

#### 2.3.1. Base of Support

**Figure 1:**
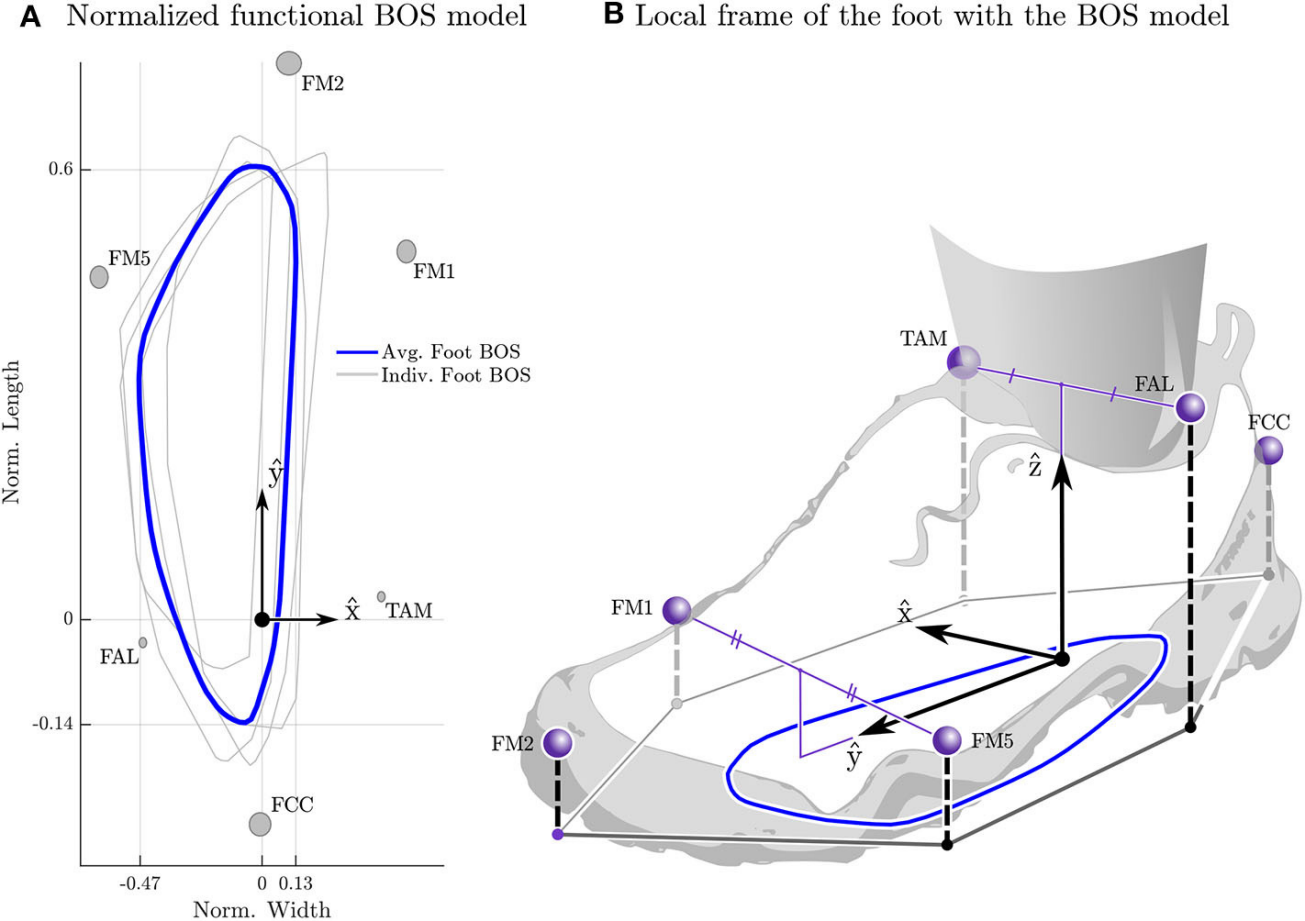
Average normalized functional base-of-support (BOS) polygon. The four BOS polygons used to build the generic functional BOS template are shown in light gray (from the left and right feet of two participants) while the average BOS polygon is shown in blue **(A)**. The BOS from each participant's right foot has been reflected about the *ŷ* and averaged with the left foot's BOS the to ensure that the final BOS polygon is symmetric. The gray ellipses are centered at the mean position for each marker resolved in the foot-fixed frame with the radius of the ellipse in the $\hat{x}$ and *ŷ* set to the standard deviation of the marker position. The *y* coordinates have been normalized by the length of the foot (*ℓ* = *ŷ* · (*r*_FM2_) − *r*_FCC_)) while the *x* coordinates has been normalized by the average width of the foot ($\text{w}=\hat{x}\cdot \left(\frac{1}{2}\left(r_{\text{TAM}}-r_{\text{FAL}}\right)+\frac{1}{2}\left(r_{\text{FM}1}-r_{\text{FM}5}\right)\right)$). The foot-fixed frame **(B)** is constructed with the origin between the two ankle markers $\frac{1}{2}\left(r_{\text{FAL}}+r_{\text{TAM}}\right)$, the *ŷ* points from $\frac{1}{2}\left(r_{\text{FAL}}+r_{\text{TAM}}\right)$ to $\frac{1}{2}\left(r_{\text{FM}1}+r_{\text{FM}5}\right)$ while the $\hat{x}$ is the component of (*r*_TAM_ − *r*_FAL_) that is perpendicular to *ŷ*, and the ẑ is given by the cross product of $\hat{x}$ and *ŷ*. This frame is then transformed so that during quiet standing the origin is on the ground plane and *ẑ* points upwards.

We define the functional BOS as the convex polygon that contains the area in which a participant can place at least half their body weight while keeping their feet flat on the ground. This polygon defines the area which the COP must stay in during the STS experiments if the participant is to complete the movement without rolling the foot excessively or taking a compensatory step. To establish the functional BOS we measured the foot movements and ground forces of two younger adult participants while they purposely moved their COP over the full functional range without taking a step. Measurements were made with shoes (one in light hiking shoes the other in Espadrilles) while standing on one foot, two feet, and also during STS trials. This data was used to create a normalized functional BOS polygon (Figure 1A) that is scaled to match each participant's left and right foot. Since both feet remain in contact with the ground throughout the STS movement we have defined the BOS as the convex hull of the ground projection of the left and right foot BOS polygons. Unless otherwise indicated we will refer to the functional BOS as simply the BOS for brevity.

The template BOS polygon was created by resolving each participant's COP profiles into a foot-fixed frame and calculating the convex hull that surrounds all COP data points in the $\hat{x}-\hat{y}$ plane (Figure 1B). Only COP points in which the foot can be considered flat and that have a normal force greater than half of the participant's body weight are included. Using a $\text{X}\left(\psi_{X}\right)-{\text{Y}}^{\prime }\left(\theta_{Y}\right)-{\text{Z}}^{\prime \prime }\left({\text{Ψ}}_{Z}\right)$ Euler-axis decomposition the foot is considered flat if w sin ψ_*X*_ ≤ 1.5cm and *ℓ* sin θ_*Y*_ ≤ 1.5cm where w and *ℓ* are the width

(1) $$\begin{array}{r} \text{w}=\hat{x}\cdot \left(\frac{1}{2}\left(r_{\text{TAM}}-r_{\text{FAL}}\right)+\frac{1}{2}\left(r_{\text{FM}1}-r_{\text{FM}5}\right)\right) \end{array}$$

and length

(2) $$\begin{array}{r} \ell =\hat{y}\cdot \left(r_{\text{FM}2}-r_{\text{FCC}}\right) \end{array}$$

of the participant's foot. We assume that together the foot pads and the sole of the shoe can compress by up to 1.5cm on one side of the foot while the unloaded side is in contact with the ground. The estimate of 1.5cm of compression comes from the fact that foot pads compress by ~ 1cm (Cavanagh, 1999; Gefen et al., 2001) during stance and the shoe sole likely compresses by 0.5cm under body weight. The template is created by normalizing each foot's BOS by the length and width of the foot and then taking the average of the four profiles from the two subjects. The average is taken by choosing a point common to all polygons as the center [in this case (−0.15, 0.3) in the left-foot normalized space], densely sampling the radius of each polygon across the common set of ray angles, and finally computing the average radius along each ray angle.

#### 2.3.2. Static Balance

Here we make use of four easily measured conditions that must be true if a participant is statically balanced: the COM_GP_ is within the BOS; the COM_GP_ and COP are closely aligned; the linear velocity of the COM (||*v*_C_||_2_) is small; and is the angular velocity (||ω_*avg*_||_2_) is small. Note that we have to use relaxed conditions (using the term *closely* rather than *exactly*, and *small* rather than *zero*) to accommodate for modeling error and the fact that even still standing is accompanied by some movement. To see if a metric has values that are consistent with static balance we compare it to the range spanned during still standing.

**Figure 2:**
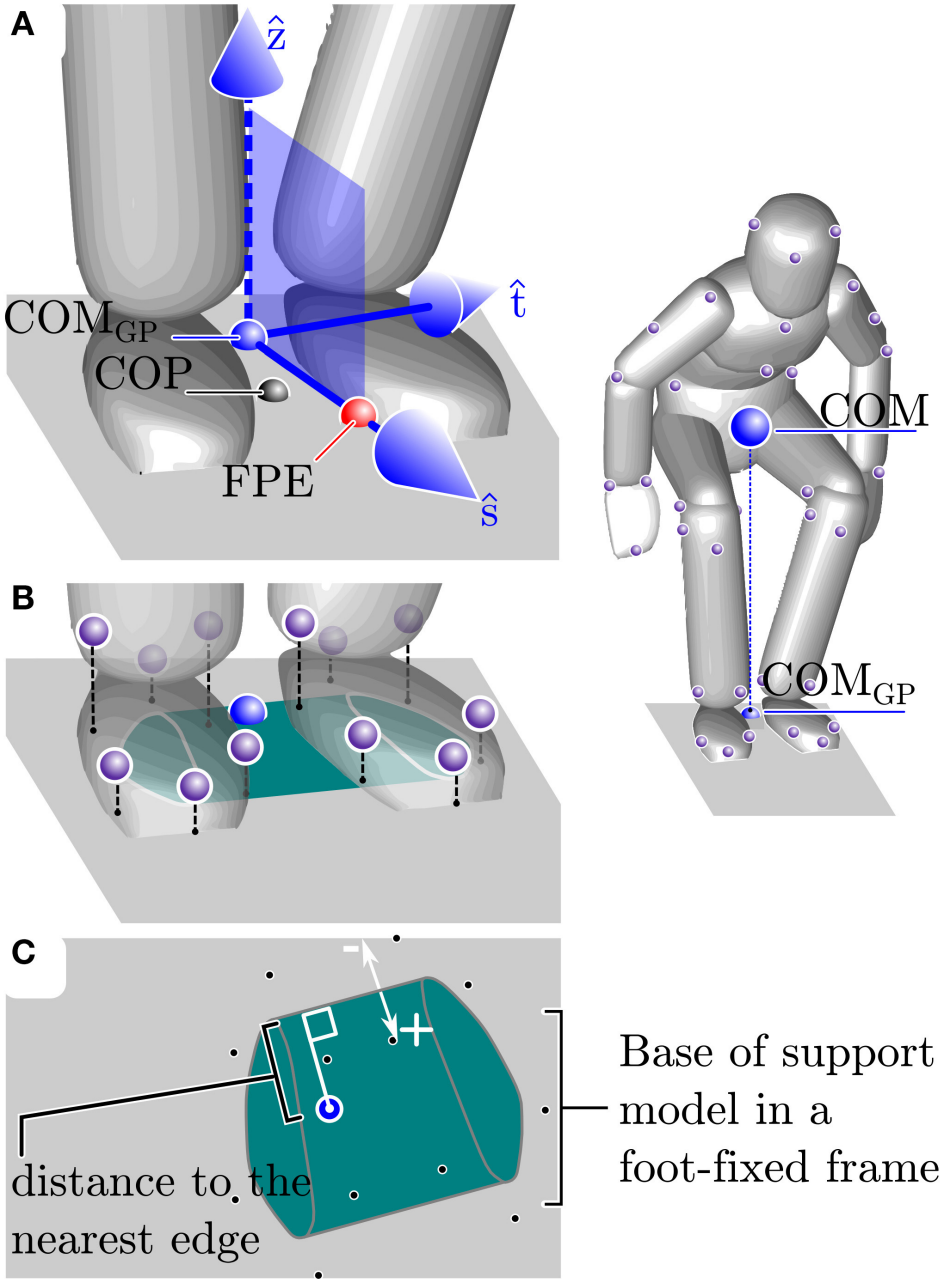
Explanation of the balance metrics. The balance metrics make use of the center-of-mass (COM), COM ground projection (COM_GP_), the center-of-pressure (COP) location, the foot-placement-estimator (FPE) location, and the base-of-support (BOS). The FPE is a model-based method (see section 2.3.3 for details) that computes the location away from the COM_GP_ along the axis *ŝ* where the COP should be placed so that the body will move into a statically balanced standing pose without any additional input **(A)**. The BOS is defined as the convex hull of the ground projection of each foot's BOS model. The BOS of each foot is defined by a polygon (see section 2.3 and Figure 1 for details) that is scaled to fit the length and width of each participant's left and right foot and is located in a foot-fixed frame **(B)**. The location of the COM and FPE are measured relative to the nearest edge of the BOS where points within the BOS are given a positive distance and points outside of the BOS have a negative distance **(C)**.

To determine if the COM_GP_ is within the BOS (Figure 2A) we measure the distance between COM_GP_ and the nearest BOS edge (_B(G)_*d*_G_). Since both feet remain in contact with the ground throughout STS, we defined the BOS as the convex-hull that enclosed the ground-projection of the scaled BOS polygons that are attached to each foot (Figure 2B). Note that a positive distance between a point and nearest edge of BOS indicates that the point is within the BOS (Figure 2C). To see how closely the COM_GP_ and the COP align we evaluate ||_G_*r*_P_||_2_ and see if the alignment is within the bound observed during still standing: since the mass distribution of the rigid body model of each participant is not perfect this distance will not go to zero even during still standing. The linear speed of the body is evaluated using ||*v*_C_||_2_ while the average angular speed of the body is evaluated using ||ω_*avg*_||_2_. Both ||*v*_C_||_2_ and ||ω_*avg*_||_2_ are compared to the bounds from our measurements of still standing.

The average angular velocity of the body ω_*avg*_ is evaluated by solving the linear system (Essén, 1993)

(3) $$\begin{array}{r} J_{C}\omega_{avg}=H_{C} \end{array}$$

where *J*_*C*_ is the moment-of-inertia of the body about the COM, and *H*_*C*_ is the angular momentum of the body about the COM. The moment of inertia *J*_*C*_ is given by

(4) $$\begin{array}{r} J_{C}=\sum_{i=0}^{\text{n}}J_{i}+m_{i}(\text{I}({\text{​}}_{\text{C}}r_{i}^{\text{T}}{\text{​}}_{\text{C}}r_{i})-({\text{​}}_{\text{C}}r_{i}{\;}_{\text{C}}r_{i}^{\text{T}})), \end{array}$$

where n is the number of bodies, *J*_*i*_ is the inertia matrix of the *i*th body at it's COM, *m*_*i*_ is the mass of the *i*th body, _C_*r*_*i*_ is the position vector from the COM of the entire system to the COM of the *i*th body, and all quantities are expressed in the inertial frame. The angular momentum vector is given by

(5) $$\begin{array}{r} H_{C}=\overset{\text{n}}{\underset{i=0}{\sum }}J_{i}\omega_{i}+{\text{​}}_{\text{C}}r_{i}\times \left(m_{i}{\text{​}}_{\text{C}}v_{i}\right), \end{array}$$

where ω_*i*_ is the angular velocity of the *i*th body, × is the cross-product, _C_*v*_*i*_ is the velocity vector from the COM of the whole system to the COM of the *i*th body, and all quantities are expressed in the inertial frame. Note that the angular velocity of the entire system is termed the average angular velocity because Equation (3) averages across the angular momentum contributions of each of the *i* bodies in the same way that *v*_C_ averages across all of the velocity contributions of each segment.

#### 2.3.3. Dynamic Balance

**Figure 3:**
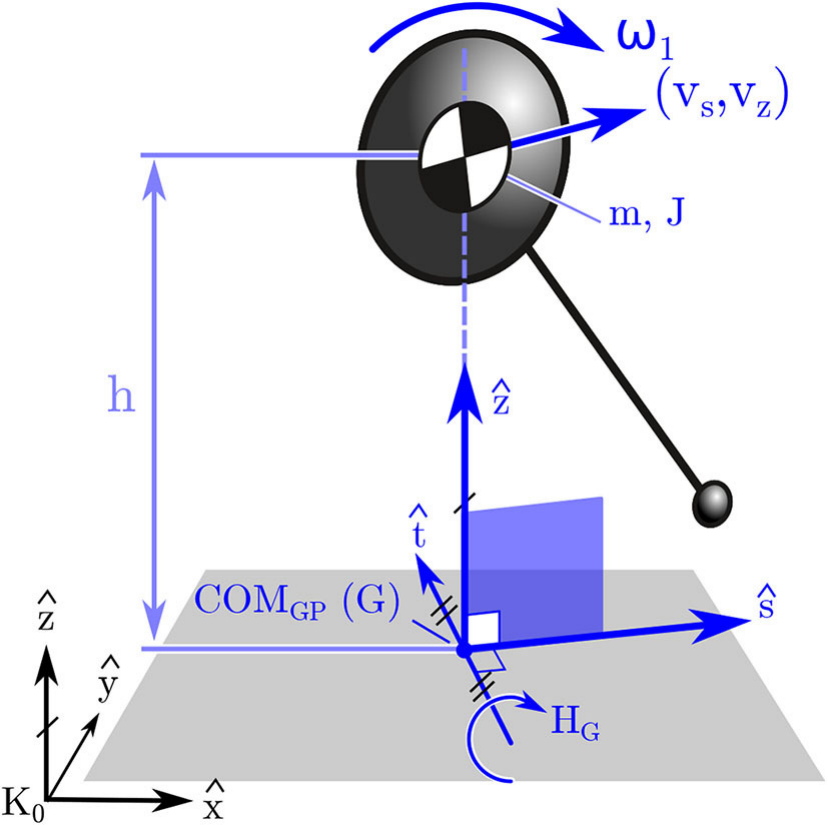
Model used to compute the FPE in 3D. The FPE is applied in 3D by first mapping the state of the participant's body to an equivalent state for a single rigid body. Next the frame centered on G is formed (described in section 2.3) and the single body state is projected onto the *ŝ* − *ẑ* frame. Finally the planar FPE location is evaluated (Wight et al., 2007) and placed along *ŝ* away from G.

Since the body may move rapidly during an STS transfer we analyzed each participant's dynamic balance throughout STS by making use of the FPE (Wight et al., 2007; Millard et al., 2009, 2012). The FPE is evaluated by first computing the state of an equivalent single-body representation of the participant (Figure 3), projecting this state onto the vertical *ŝ*-*ẑ* plane, and finally by applying the planar FPE (Wight et al., 2007) to this projected state. Here we use the FPE to measure four quantities related to dynamic balance: the error incurred when projecting the body's state onto the *ŝ*-*ẑ*, the size of the dynamic balance margin; the distance between the COP and the FPE in $\hat{t}$ (which causes turning); the distance between the COP and the FPE in *ŝ* (which accelerates the body forwards and backwards).

**Figure 4:**
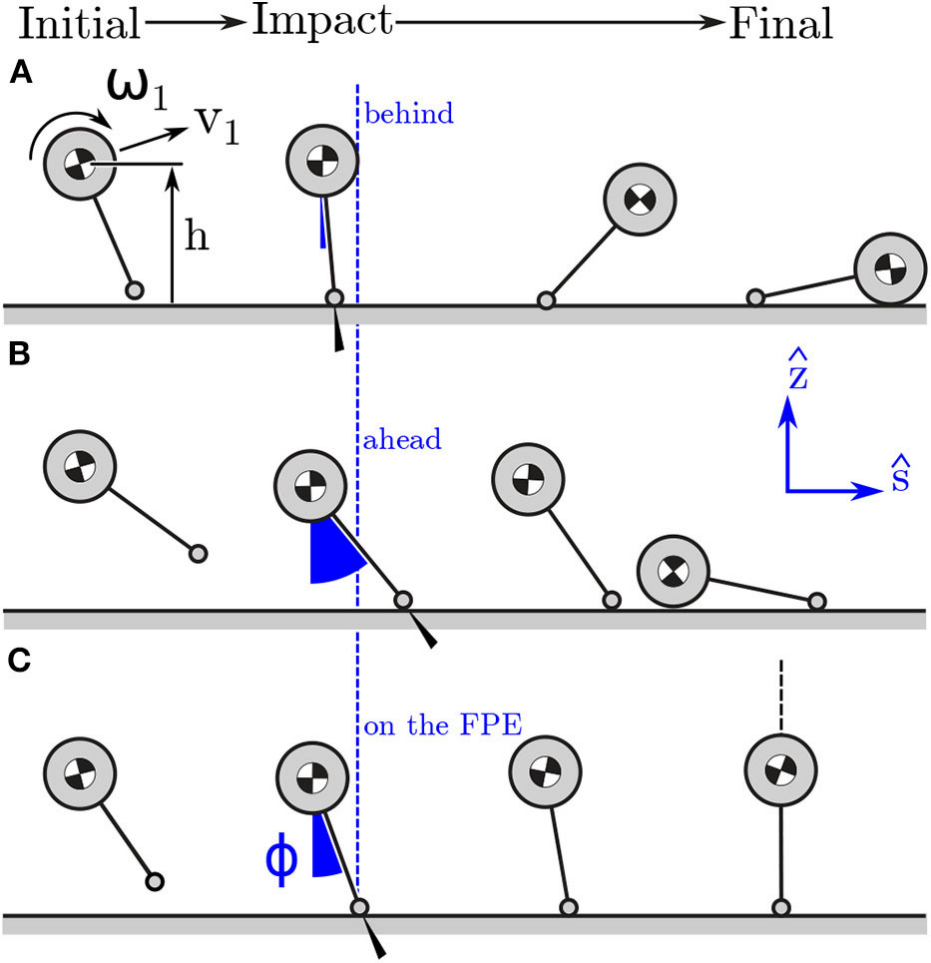
Model stepping behind **(A)**, ahead, **(B)** and on **(C)** the FPE in the *ŝ* − *ẑ* plane. Stepping behind the FPE location will mean that the post-contact kinetic energy of the model cannot be exchanged for potential energy: the model falls forwards. In contrast, if the model has the same initial conditions but steps far ahead of the FPE the model will not have enough post-contact kinetic energy to rotate passively over its foot: the model falls backwards. Stepping on the FPE location will ensure that the post-contact kinetic energy of the model exactly equals the potential energy the model will gain when it is standing over its contact point: in this case the model transitions to a quasi-stable standing position.

The FPE is most accurate in the special case when the state of the body can be projected onto the *ŝ*-*ẑ* plane (Figure 3) without loss of information. The *ŝ*-*ẑ* is a vertical plane with its origin at COM_GP_. The direction vectors are derived using the whole-body angular momentum vector *H*_*G*_. The direction vector $\hat{t}$ (Figure 3) is in the direction of horizontal component of *H*_*G*_, and the direction vector *ŝ* is defined using the cross-product of $\hat{t}$ with *ẑ*: if *H*_*G*_ has no component in *ẑ* it can be projected onto the *ŝ*-*ẑ* plane without loss of information. The vector *H*_*G*_ will have no component in the *ẑ* if ω_*avg*_ · *ẑ* = 0. Though this ideal has been satisfied to small tolerances when applied to walking and jumping (Millard et al., 2009, 2012), it is not clear if this condition will be met during STS. Therefore, we examined the vertical component of the average angular velocity vector: if ω_*avg*_ · *ẑ* ≤ ϵ, where ϵ is a bound defined using still standing data, it means the movements of the participant are consistent with the assumption used to extend the planar FPE model to 3D. It is important to note that the condition that ω_*avg*_ · *ẑ* = 0 must be met exactly by the simple model otherwise the trajectory of the pendulum will exit the *ŝ* − *ẑ* plane and the pendulum's motion will not terminate in a quasi-stable standing pose (Figure 4C). In contrast, when the FPE is applied to a multibody system, such as a human, small values of ω_*avg*_ · *ẑ* can be tolerated since the extra degrees-of-freedom of the body can be used to compensate for the trajectory errors introduced by non-zero values of ω_*avg*_ · *ẑ*.

The FPE is a location in the direction *ŝ* away from *r*_G_: steps that are shorter than the FPE will cause the model to fall forwards (Figure 4A), steps that are longer will cause it to fall backwards (Figure 4B), steps that are exactly on the FPE will allow the model to come to rest passively as the COM passes over the COP (Figure 4C). Since steps taken in *ŝ* will preserve the direction of *H*_G, 1_ to *H*_G, 2_ this axis is named the straight-step direction. In contrast, steps taken in $\hat{t}$ will change the direction of *H*_G, 1_ to *H*_G, 2_ and cause a turn, and so we name $\hat{t}$ the turning direction. What follows is a derivation of the planar FPE (Wight et al., 2007) so that the dynamic balance metrics computed using the FPE are clear. We begin by assuming that momentum is conserved about the model's contact point

(6) $$\begin{array}{r} H_{\text{G},1}=H_{\text{G},2} \end{array}$$

before (indicated using _1_) and after (indicated using _2_) the contact is made. This expression can be expanded (using *sϕ* for sin ϕ and *cϕ* for cos ϕ for brevity) by making use of the assumption that prior to contact the linear (*v*_*s*, 1_ = *v*_C_ · *ŝ* and *v*_*z*, 1_ = *v*_C_ · *ẑ*) and angular velocity ($\omega_{1}=\omega_{avg}\cdot \hat{t}$) of the model are uncoupled but after contact the model moves in a pure rotation (ω_2_) about the contact point on a leg of fixed length *ℓ*

(7) $$\begin{array}{r} m\ell \left(v_{s,1}c\phi +v_{z,1}s\phi \right)+J\omega_{1}=\left(m\ell^{2}+J\right)\omega_{2} \end{array}$$

where

(8) $$\begin{array}{r} J={\hat{t}}^{\text{T}}J_{C}\hat{t} \end{array}$$

is the moment of inertia of the COM about $\hat{t}$. For a candidate ϕ it is assumed that the leg length is constant *after* contact, but before contact it is long enough to reach the ground

(9) $$\begin{array}{r} \ell =\frac{h}{c\phi } \end{array}$$

where *h* = *r*_C_ · *ẑ* is the height of the COM. It is worth noting that the leg length is the distance between the COM and COP which may not correspond to the physical leg length of a human or robot. After substituting Equation (9) into (7) we can isolate ω_2_

(10) $$\begin{array}{r} \omega_{2}=\frac{mh\left(v_{s,1}c\phi +v_{z,1}s\phi \right)c\phi +J\omega_{1}c^{2}\phi }{mh^{2}+Jc^{2}\phi }. \end{array}$$

The FPE is defined as the contact location such that the model comes to rest just as its COM_GP_ comes into alignment with its COP. This means that the kinetic energy of the model just after contact must be exactly equal to the potential energy the model will gain as its COM moves from an initial height of *h* to *ℓ* as it rotates forwards. Since the model is in a pure rotation after contact we arrive at the following energy balance that must hold if ϕ points to the FPE location

(11) $$\begin{array}{r} \frac{1}{2}\left(J+m\ell^{2}\right){\omega_{2}}^{2}=mg\left(\ell -h\right). \end{array}$$

Substituting Equation (10) into Equation (11) and simplifying yields a non-linear function

(12) $$\begin{array}{r} f=\frac{{\left(mh\left(v_{s,1}c\phi +v_{z,1}s\phi \right)c\phi +J\omega_{1}c^{2}\phi \right)}^{2}}{mh^{2}+Jc^{2}\phi }+2mgh\;c\phi \left(c\phi -1\right) \end{array}$$

that evaluates to 0 when ϕ is at the angle that dynamically balances the model. In practice the value of ϕ that satisfies the constraint *f* = 0 is solved numerically using the bisection method to get close to the solution and Newton's method to polish ϕ to high precision. Trigonometry is then used to find the vector from the inertial frame to point F

(13) $$\begin{array}{r} r_{\text{F}}=r_{\text{G}}+\left(h\tan\phi \right)ŝ. \end{array}$$

To apply the FPE to an STS transfer, this set of calculations is repeated for every recorded time sample. Since the single-body equivalent state of the participant and the FPE is re-evaluated at every time sample the assumptions of the method (*J* is constant, *ℓ* is constant, and the sum of kinetic and potential energy is constant) introduce small amounts of error in ϕ (at most 1.47° for the younger adults and 3.58° for the older adults, see Supplementary Material for details).

The difference between the model's point contact and the comparatively large BOS provided by a typical human foot affect how the FPE is interpreted. The closest physical analog to the model's point contact is the COP location of the BOS: in both cases moments (ignoring spin friction) taken about this point vanish to zero. Thus, if the FPE is within the BOS the participant can remain dynamically balanced by matching the COP location with the FPE. In contrast, if the FPE is outside of the BOS a participant will have to take a physical step that captures the FPE within the BOS in order to eventually transition to a state of static balance. For STS transfers participants keep their feet fixed on the ground throughout the movement and so we can define the *dynamic balance margin* as the displacement between the nearest BOS edge and the FPE (_B(F)_*d*_F_). As before a positive sign indicates that point F is within the BOS.

Even though participants are restricted from moving their feet, they can still modulate the location of their COP relative to the FPE: modulating the COP location along the $\hat{t}$ axis will cause the model to rotate about the *ŝ* axis and turn; varying the COP location in *ŝ* will cause the model to fall forwards or backwards (as illustrated in Figure 4). Since turning is not a part of the task, we checked the displacement from the COP and the FPE location in $\hat{t}$ (${\;}_{\text{P}}r_{\text{F}}\cdot \hat{t}$) to see how well participants are able to maintain the direction of travel. To see if participants are modulating their COP in *ŝ*, either to help propel them out of the stool or to slow down, we also measure the displacement from the COP to the FPE location in *ŝ* (_P_*r*_F_ · *ŝ*).

#### 2.3.4. Balance Metrics

In summary, we calculated the following static and dynamic balance metrics:

- Static balance metrics: (1) displacement from the BOS edge to the COM_GP_ (_B(G)_*d*_G_); (2) distance between COM_GP_ and COP (||_G_*r*_P_||); (3) COM speed (||*v*_C_||_2_); (4) whole-body average angular speed ||ω_*avg*_||_2_
- Dynamic balance metrics: (1) angular velocity about the vertical axis (ω_*avg*_ · *ẑ*); (2) displacement from the BOS to the FPE (_B(F)_*d*_F_) i.e. the dynamic balance margin; (3) distance from the COP to the FPE location in turning direction (${\;}_{\text{P}}r_{\text{F}}\cdot \hat{t}$); (4) distance from the COP to the FPE in straight-step direction (_P_*r*_F_ · *ŝ*).

This set of static and dynamic balance measures allows us to create a rich picture of how each of the participants coordinate their body and the forces acting on it to execute an STS transfer. We expect that participants who are frail, or afraid, will execute STS transfers while staying statically balanced or nearly so. We expect to see that participants who struggle with balance will allow the FPE location to approach the edge of their BOS, while participants who balance effectively can keep their FPE within the BOS and further from the edges. Since the STS transfer does not involve spinning or turning we expect to see all participants maintain small values of ω_*avg*_ · *ẑ* and keep their COP as close as possible to the FPE in $\hat{t}$. We expect that older adults who are confident in their movements but struggle to get out of the stool may bias their COP behind of the FPE in the *ŝ* direction to help propel them forwards out of the stool. Finally, we expect the older adults to display larger variation than the younger adults in all of the metrics.

### 2.4. Statistics

The 8 balance metrics taken at seat-off, generally considered the most unstable moment during STS, was used for analysis (see Supplementary Table 1 for more information on stance). In addition, we analyzed total STS duration and duration from seat-off to stance. To assess differences in performance between groups, values were averaged over STS repetitions per participant; for differences in variability we took the range over repetitions measured for each individual. As group size was limited, we used non-parametric tests. Primary analysis tested for differences between young and older participant groups in the Side condition using unpaired Wilcoxon rank sum test. Secondary analysis tested for differences between arm conditions Side and Chest using paired Wilcoxon signed rank tests per age group. Reported values represent median and interquartile ranges (from 25 to 75%). Significance was set at *p* < 0.05 and statistical analyses were performed in Matlab (version 2019a, Natick, MA, USA).

## 3. Results

**Figure 5:**
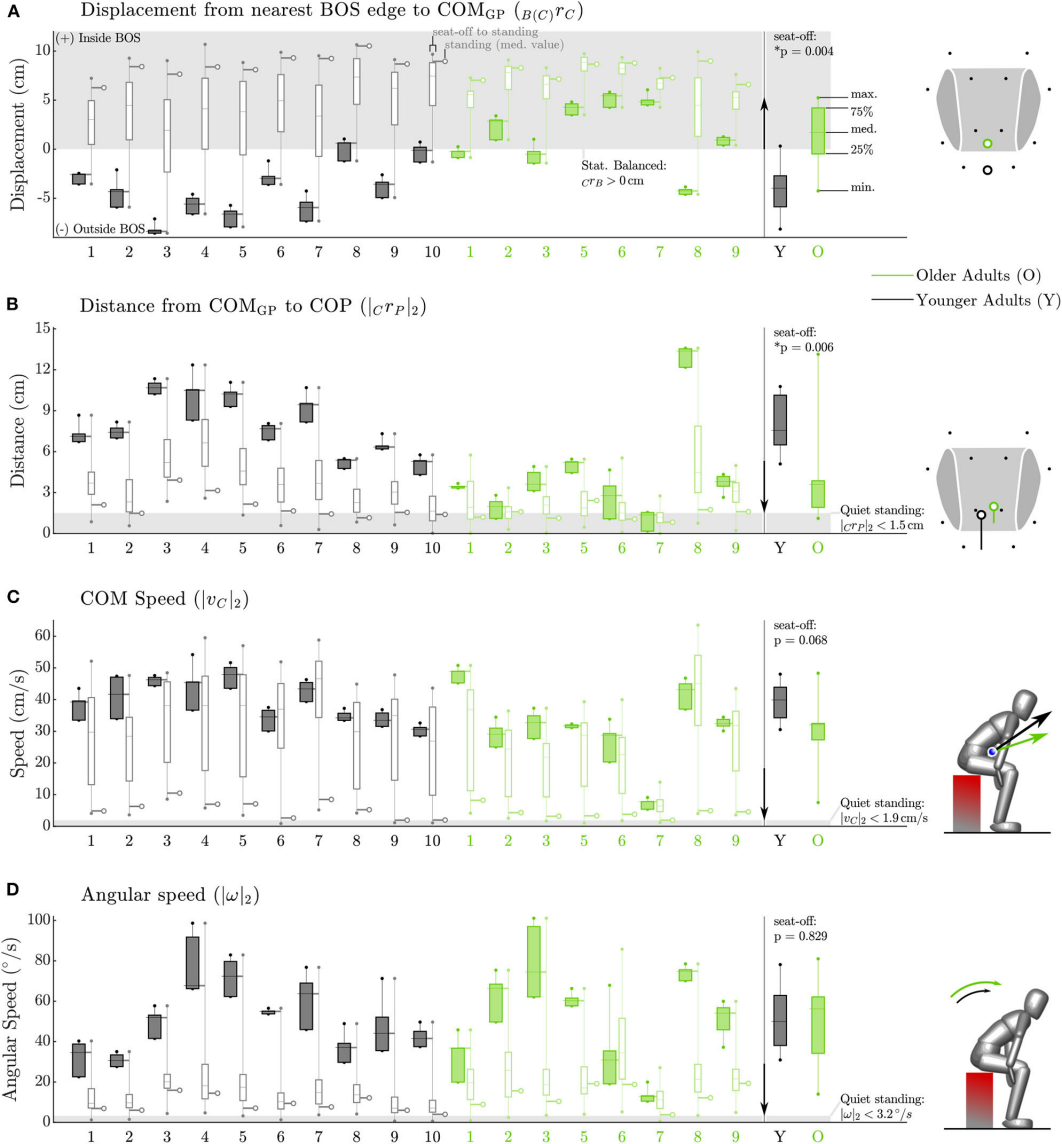
Static balance metrics for the Side condition. Data for each participant appears on the left while group data at seat-off appears on the right. The older participants (green) keep their COM_GP_ within their BOS from seat-off to standing while nearly all of the younger participants (black) begin seat-off with their COM_GP_ outside of the BOS **(A)**. The older adults keep their COP closer to their COM_GP_ than do younger adults **(B)**. The older adults move their COM more slowly between seat-off and standing than do younger adults **(C)**. The average angular speed of both the older and younger participants is quite high at seat-off **(D)**. The gray box indicates the static balance region in **(A)** and values measured during quiet standing in subplots **(B–D)**. The arrow between the individual and group data indicates the direction toward more conservative static balance.

The four static balance measures all indicated that the older adults stay closer to being statically balanced than the younger participants between seat-off and standing (Figure 5). At seat-off, most of the older adults had their COM_GP_ 1.7 [4.7] cm inside the BOS and kept it there throughout the movement to stance, while nearly all of the younger participants began seat-off with their COM_GP_ outside of their BOS (−4.0 [3.2] cm, *p* = 0.004, Figure 5A). While both groups closely aligned their COM_GP_ and COP at standing, the older adults began seat-off with a smaller distance of 3.6 [2.0] cm compared to 7.6 [3.6] cm for the younger participants (*p* = 0.006, Figure 5B). The maximum speed of the COM of the older participants tended to be lower throughout the movement than their younger counterparts, with 32.1 [5.2] compared to 39.9 [9.6] cm/s at seat-off (*p* = 0.068, Figure 5C). There was no significant difference between older and younger adults in the total STS duration (1.9 [0.9] vs. 1.7 [0.2] s, *p* = 0.315) but there might be a trend of older adults requiring more time to move from seat-off to standing (1.0 [0.3] vs. 0.8 [0.1] s, *p* = 0.122). Both older and younger adults had significant angular speeds during STS (Figure 5D), with values as high as 56.2 [28.0] and 50.0 [24.8] °/s at seat-off (*p* = 0.829) and lower speeds over the entire movement (18.9 [11.5] and 12.0 [10.2] °/s). There was no detectable difference in within-subject variability (as defined in section 2.4) between the older and younger adults in the duration of the movement nor in any of the four static balance measures (*p* = 0.12–0.95 see Supplementary Table 2).

**Figure 6:**
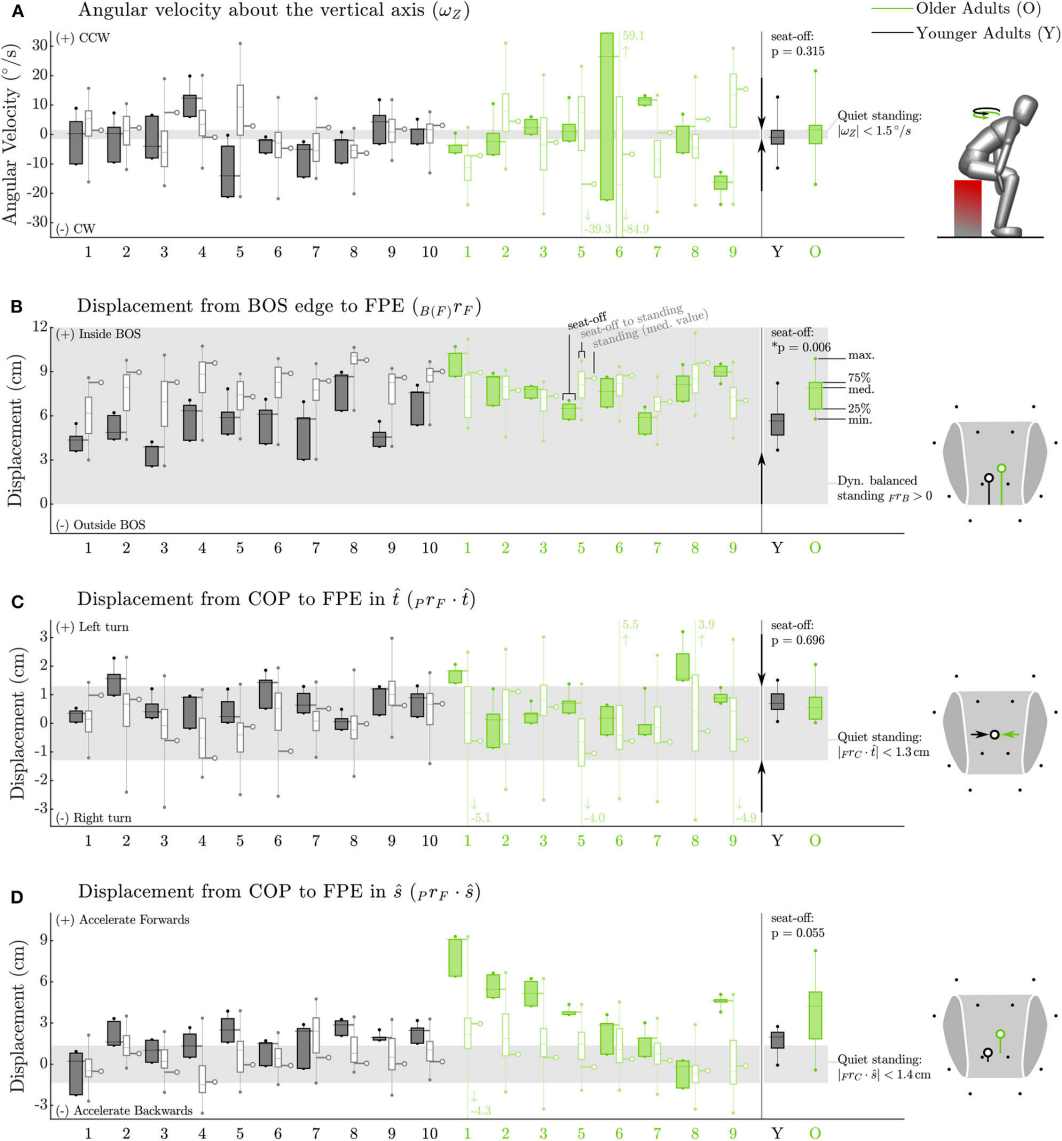
Dynamic balance metrics for the Side condition. Data for each participant appears on the left while group data at seat-off appears on the right. The FPE method assumes a small angular velocity about the vertical axis. While this assumption is reasonable for most participants it is not appropriate for all participants, particularly O6 **(A)**. Both groups (older adults in green, younger adults in black) keep the FPE similarly well within the BOS (shown in gray) between seat-off and standing and **(B)**. Both groups display a tight control of the COP relative to the FPE location in the turning direction $\hat{t}$ **(C)** indicating that neither group is turning on average between seat-off and standing. Some of the older adults bias COP behind the FPE at seat-off **(D)** presumably as a strategy to help propel them off of the stool. The arrows between the individual and group data indicate the direction toward a state of more conservative dynamic balance. No arrow appears in the plot of _P_*r*_F_ · *ŝ* **(D)** as deviations in *ŝ* are permitted (and thus indicate individual preference) provided _B(F)_*d*_F_ is positive.

The older adults appeared to control their balance similarly well as the younger adults, while maintaining a larger dynamic balance margin (Figure 6B). Both groups almost maintained low angular velocities about the vertical axis (Figure 6A), with values of ω_*avg*_ · *ẑ* as small as 1.62 [6.2] and −0.95 [4.6] °/s at seat-off (*p* = 0.315) and −2.3 [18.5] and −0.035 [7.2] °/s on average throughout the movement. This indicates that for nearly all participants the FPE should be accurate, except for participant O6 who moved with values of ω_*avg*_ · *ẑ* that were much larger than other participants at seat-off (26.5 [56.6] °/s) and throughout the movement (−17.1 [54.9] °/s). Both young and older adults kept the FPE well within the BOS between seat-off and standing, with the older adults maintaining larger dynamic balance margins than younger adults (7.9 [1.8] vs. 5.7 [1.4] cm *p* = 0.006) at seat-off (Figure 6B). In addition, both groups maintained the direction of travel, having a distance between FPE and COP in the $\hat{t}$ direction close to zero with a narrow spread: 0.6 [0.8] cm for the older vs. 0.7 [0.5] cm for the younger adults (*p* = 0.696), Figure 6C). In the *ŝ* direction, some of the older adults showed a preference for beginning seat-off with a larger distance between the FPE and COP than the younger participants (4.2 [3.4] vs. 2.0 [1.2] cm, *p* = 0.055, Figure 6D) presumably as a strategy to help propel them out of a seated position. No differences were found in variability between the older and younger adults for any of the dynamic balance measures (*p* = 0.38–0.97 see Supplementary Table 2).

**Figure 7:**
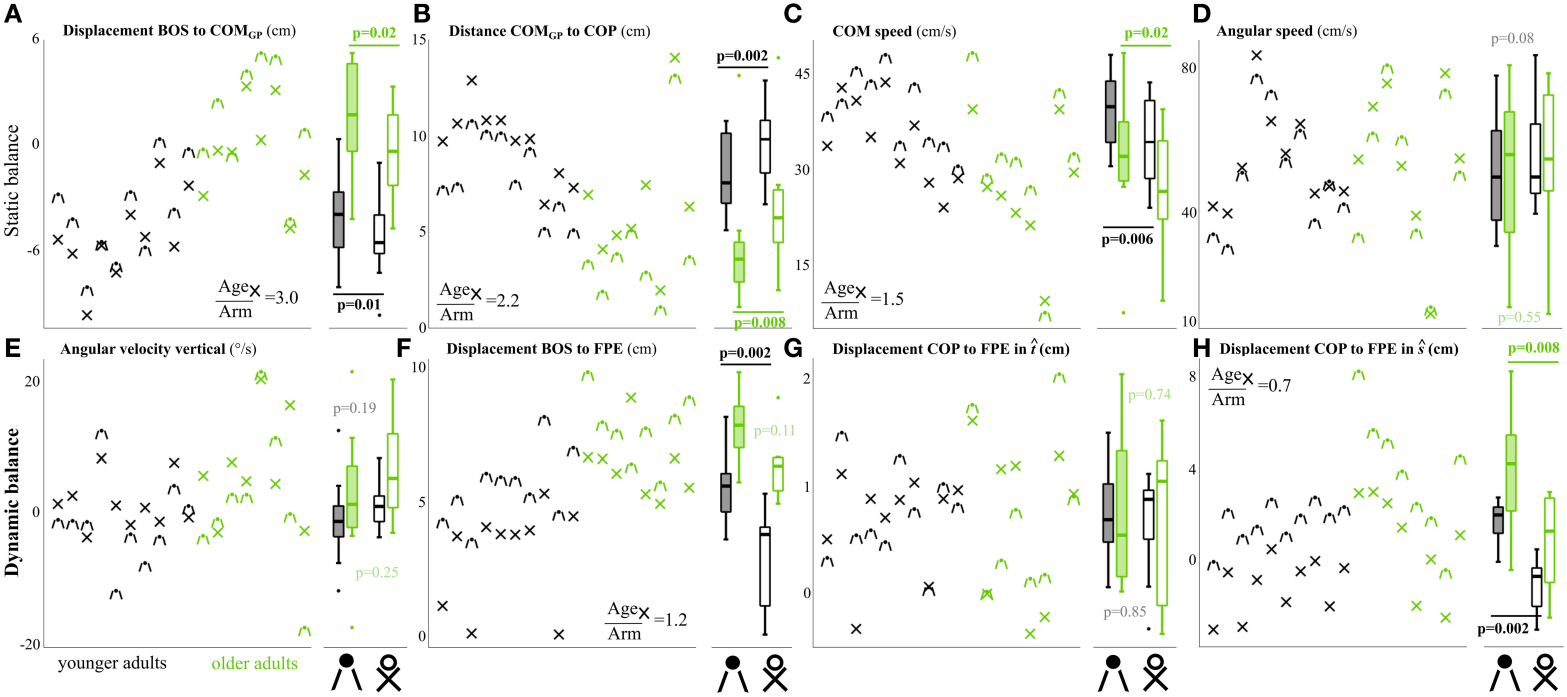
Comparison between Side and Chest conditions for all static **(A–D)**, and dynamic **(E–H)** balance metrics. Individual median data points are shown for younger (black) and older adults (green). Conditions are shown with x-mark for Chest condition and dot (median value) with hanging side arms for Side condition. Although most variables are affected by arm condition, the differences in balance control between the groups are preserved between conditions. *P*-values from the paired non-parametric sign-rank tests are given comparing effect of condition per group, in bold when significant (p < 0.05). In addition, the ratio of age effect for the Chest condition and arms condition (*O*^*C*^ − *Y*^*C*^)/((*O*^*C*^ + *Y*^*C*^) − (*O*^*S*^ + *Y*^*S*^)) (where *O* and *Y* stand for older and younger, and *C* and *S* stands for Chest and Side) is indicated for the affected variables. Box plots indicate the median, 25^th^ and 75^th^ percentiles of the group data, and the whiskers the extreme values that are not considered outliers (plotted as dots).

Several static and dynamic balance metrics were affected by the arm conditions, although the effect of age was dominant and preserved between conditions (Figure 7). While both total duration and duration from seat-off to stance were not affected by the position of arms in either group (*p* > 0.195), both groups were further from being statically stable at seat-off with arms crossed at their chest compared with arms at the side (Figure 7). Specifically, both groups kept their COM_GP_ further from the center of the BOS (*p* < 0.016), as well as from the COP (*p* < 0.008), but they did have lower COM speed (*p* < 0.016). In addition, both groups tended to decrease their dynamic balance margin by placing their FPE closer to the BOS edge (Y: *p* = 0.002, O: *p* = 0.109), as well as closer to the COP in the *ŝ* direction (*p* < 0.002), thus reducing the forward propelling strategy. As these differences between arm conditions were in the same direction for both age groups, the differences in balance control between age groups are dominant over the arm effect. The effect of age at the chest condition was 1.5–3.0 times as large for the static balance variables that were affected by arms condition and 1.2 times for the FPE to BOS (Figure 7). Only the effect of arms condition on the FPE-COP excursions in *ŝ* was larger compared with the age effect (0.7 times, see Supplementary Material).

## 4. Discussion

As STS performance degrades, many older adults suffer injuries due to falls (Rapp et al., 2012; Pozaic et al., 2016; van Schooten et al., 2017). Previous investigations into STS balance have examined quantities that do not directly measure the conditions necessary for balance but instead quantities that correlate with performance: including STS duration, COM kinematics and COP to ankle position at seat-off (Moxley Scarborough et al., 1999; Åberg et al., 2010; Akram and McIlroy, 2011; Fujimoto and Chou, 2014). We began our balance assessment by calculating how close each participant is to being statically balanced during their movement: the displacement between COM_GP_ and BOS, the distance between COM_GP_ and COP, the speed of the COM, and the average angular speed of the whole body at seat-off. In addition, we assessed dynamic balance by applying the FPE, allowing us to define an STS as being balanced if the FPE remained within the BOS throughout STS. In addition, we analyzed the distance between FPE and COP in both straight step and turning directions.

Although the FPE allows us to take both linear and angular momentum into account, it comes at the cost of more involved modeling and mathematics. Therefore, we analyzed the necessity and applicability of using the FPE by evaluating the angular speed as well as the assumption of small angular velocity about the vertical axis. Regarding the necessity, we found that all participants moved with a large whole-body angular speed, especially at seat-off. Clearly it is important to use a balance metric, like the FPE, that takes angular velocity into account. Regarding the applicability, we found that the assumption of small ω_*avg*_ · *ẑ* was well met by nearly all of the participants except Y5, O6, and O9. This is in contrast to previous work in which the FPE was used to study walking motions Millard et al. (2009); Bruijn et al. (2013) where the condition ω_*avg*_ · *ẑ* = 0 is satisfied to a small tolerance. Though the values reported in Figure 6 may seem large, neither Y5 nor O9 visibly struggled with balance during the STS trials presumably because they could compensate for a non-zero ω_*avg*_ · *ẑ* after seat-off. In contrast, O6 did visibly struggle with balance during the STS trials. Due to O6's unique pathology (hemiplegia) in our participant group we re-ran the analysis excluding O6 and found no changes to our results. It is striking that the participant who most visibly struggled with balance was also the only participant with large and highly variable values of ω_*avg*_ · *ẑ*. In all other respects the static and dynamic balance metrics of O6 (Figures 5, 6) are unremarkable: it is as if O6 has retained all faculties to balance except to regulate ω_*avg*_ · *ẑ* to small values. A failure to control ω_*avg*_ · *ẑ* has consequences beyond the applicability of the FPE: it will make it difficult to control the direction of travel which has a clear impact on maintaining balance. In the future it will be valuable to study ω_*avg*_ · *ẑ* in more detail so that it is clear what range is associated with typical movements and what ranges might be indicative of a balance pathology. Further it will be important to determine if others who struggle with balance also exhibit large variations in ω_*avg*_ · *ẑ* or if this is a problem specific to people (such as O6) who have an asymmetric pathology like hemiplegia.

Our approach of analyzing both static and dynamic balance yielded a surprise: although the older adults stay closer to being statically balanced than the younger adults, supporting our first hypothesis, both groups maintained similar dynamic balance margins, refuting our second hypothesis of reduced dynamic balance in older adults. In addition, there was no indication that older adults display more variability in the execution of STS refuting our final hypothesis. As such, the results of the static balance analysis generally echo what has been previously found: older adults are slower, keep their COM_GP_ more anterior, and have an increased rising duration compared to younger participants. It has been reported that older adults place their COM and COP further forward at seat-off than younger adults, and more so in those with previous falls, impairments or STS difficulty (Schultz et al., 1992; Aissaoui and Dansereau, 1999; Papa and Cappozzo, 2000; Chen et al., 2013; Fujimoto and Chou, 2014). In addition, older adults exhibited lower trunk or body speed, especially those with higher frailty level, fear of falling or failed STS attempts (Riley et al., 1997; Kouta and Shinkoda, 2008; Åberg et al., 2010; Ganea et al., 2011), although compensatory increases in trunk flexion have been reported as well (Papa and Cappozzo, 2000). In accordance with the lower body speed, older adults tended to require more time for the most demanding STS phase, to move from seat-off to stance, aligning with the general notion that they are slower to rise especially when more frail or impaired (Ganea et al., 2011). However, our data shows that while older adults execute STS more slowly and conservative as noted in literature, they are dynamically balancing as well as the younger participants with similar levels of variability between repetitions. Thus, the changes in performance with age do not seem to reflect impaired balance control, but are likely a compensatory mechanism for reduced physical ability or reduced confidence.

Both the static and dynamic balance analysis depend on knowing the geometry of the BOS. To make our analysis as accurate as possible we have developed a BOS model of a shod foot. Since the BOS polygon of the foot has been fitted to data of two younger adults (Figure 1) it may not accurately represent every participant. It is important to note that the definition of the BOS does affect some of our results. Using an alternative BOS model (the convex hull of the ground projection of the motion capture markers attached to the feet) the differences between younger and older adults in _B(F)_*d*_F_ (Figure 6B) are no longer significant (*p* = 0.633), though both groups still maintain positive dynamic balance margins. Though other numerical results change, no other statistical differences are affected by the change in BOS model. We have chosen to present the results obtained using the functional BOS model because this model should be more accurate in principle than the simpler model. In addition the range spanned by _B(F)_*d*_F_ between the 25th–75th percentiles is smaller using the functional BOS in comparison to the simpler alternative model: [1.8] vs. [3.8] for the older adults and [1.4] vs. [1.8] for the younger adults. The reduction in the span from the 25th–75th percentile indicates that the BOS model is removing some of the systematic variations in _B(F)_*d*_F_ that participants are making to accommodate for the size and shape of their feet. In the future we hope to make the same detailed measurements of the BOS across a larger range of participants to see how the functional BOS varies from person to person.

This study introduced a combination of clustering and adaptive thresholding that allowed us to identify different transitions in the movement, including seat-off and standing. As some of our older adults are quite variable between STS movements, including some sit-back failures, a single threshold did not work well within, let alone between, participants. Although the segmentation algorithm is a little elaborate the alternative has drawbacks: manually identifying these events would have been subjective and not reproducible. We expect similar results would have been obtained had we manually segmented every movement as our analysis focused at the moment of seat-off which can be accurately identified using the force plate data. We also confirmed using a few of test cases that manually identifying the transition to standing yields similar results to the automatic segmentation routine. In contrast, consistently identifying the time of STS initiation and standing manually would be challenging. We hope others find this approach useful to segment movement data while adapting to each participant's characteristics.

In addition, we have also found that the while the arm conditions of the STS transfer affect the movement, many similar observations were made in the Chest compared to the more natural Side condition. Despite this similarity, the differences between the arm conditions are large enough that we feel that STS should be assessed using conditions that are as natural as possible: otherwise trends might be observed which are due to the unnatural lack of arm motion rather than an underlying condition. It should be noted that other conditions might be even more natural to people, such as providing support using the legs or arm rests. The effect of different types of assistance on the STS movement and especially balance will be part of future research.

The main limitation of this study is the relatively small size of the groups that are included. It is possible that differences in dynamic balance control could therefore not be detected, but we were able to detect differences in static balance control between older and younger participants. In addition, we were not able to include older adults with known STS difficulties; however, the clinical metrics show that we have a rather heterogeneous sample, ranging from those who are managing well to those with reduced physical ability. This was reflected by some older adults showing observable difficulty with getting up, resulting in a few failed attempts, as is represented by lower SPPB values. Regardless, as some older adults performed in the range as younger adults, this could have masked differences in dynamic balance and variability that might be characteristics for more frail older adults. As such, our future research will be directed to gathering data in more frail older adults, including those who are (more) dependent on assistance, and also focus on failed STS attempts.

We have made several important contributions in this work: we have analyzed the conditions for static and dynamic balance during STS by applying the FPE for the first time to STS, and by using a geometric model of the BOS. While few studies have used a point of convenience, such as the ankle, as a reference point before (Schultz et al., 1992; Moxley Scarborough et al., 1999; Papa and Cappozzo, 2000; Fujimoto and Chou, 2014), the conditions for even static balance cannot be evaluated without a BOS model. Our work fills a void in the literature since existing studies have not analyzed all of the quantities necessary to assess balance directly but instead have focus on a few isolated metrics: STS duration, COM speed, trunk movement, COM-ankle distance, COP-ankle distance (Moxley Scarborough et al., 1999; Akram and McIlroy, 2011; Jeyasurya et al., 2013; Fujimoto and Chou, 2014). Together, our approach allowed us to show that quantities measured in existing literature that differ between younger and older adults (Aissaoui and Dansereau, 1999; Moxley Scarborough et al., 1999; Janssen et al., 2002; Åberg et al., 2010; Akram and McIlroy, 2011; Fujimoto and Chou, 2014; Millor et al., 2014; Boukadida et al., 2015) may not actually pertain to reduced balance control in the latter group. As is evidenced in our data, it is possible to execute an STS, slowly or quickly, with a lot of COP movement or a little, all while displaying a fine control of dynamic balance.

This work provides an important but first step in exploring balance during STS in older adults. As with most metrics that have suggested to quantify balance during movements, further validation to demonstrate how these static and dynamic balance metrics actually relate to falls is needed. Therefore, this analysis should be repeated in older adults that are prone to falling, have a fear of falling, are more frail or have different impairments. More specifically, it would be interesting to contrast the static and dynamic balance values for successful versus failed STS attempts, including sit-back, side-step, and step-forward attempts, and simulated falls. To simulate falls, an important and open question has yet to be answered: how do older adults actually fall during sit-to-stand and stand-to-sit.

## 5. Conclusion

In this work we have shown that while older adults execute STS more slowly and stay closer to being statically balanced than younger adults, they are dynamically balancing as well as the younger participants with similar levels of variability. Our analysis of static and dynamic balance indicates that the reason for this difference is not due to a reduced sense of balance. Thus, the presented approach of using the model-based dynamic balance metric FPE as well as expressing metrics relative to individual's BOS, allows us to distinguish between STS movement (such as duration and COM speed) and balance. Future research is needed to see how the patterns of static and dynamic balance change between balanced and unbalanced motion, and between people who are prone to falling from those who move safely.

## Data Availability Statement

The data supporting the conclusions of this article will be made available by the authors, without undue reservation.

## Ethics Statement

The studies involving human participants were reviewed and approved by the IRB of the medical faculty of Heidelberg University. The participants provided their written informed consent to participate in this study.

## Author Contributions

LS, CW, and KM designed the experiment. LS and CW collected the data and recruited the participants. MM and LS processed and analyzed the data, interpreted the data, wrote the manuscript, and generated the figures and tables. All authors provided the critical feedback on the manuscript.

## Conflict of Interest

The authors declare that the research was conducted in the absence of any commercial or financial relationships that could be construed as a potential conflict of interest.
