# Supplementary material for: Slow but Steady: Similar Sit-to-Stand Balance at Seat-Off in Older vs. Younger Adults
Source: Front Sports Act Living. 2020 Oct 26;2:548174. doi: 10.3389/fspor.2020.548174 (PMC7739623; doi:10.3389/fspor.2020.548174)
Supplement: Supplementary file 1 [file Data_Sheet_1.pdf]

Supplementary Material

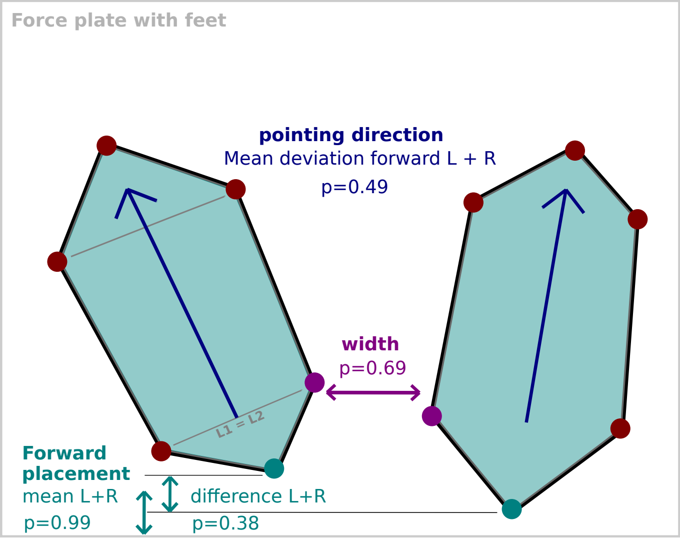

Figure S1. Similar foot placement between younger and older adults. As most metrics in this paper are related to individual's base of support (BOS), we evaluated whether foot placement was comparable between groups. The width between the feet, the degree of pointing forward, the forward placement and feet symmetry of forward placement were calculated for both the younger and older participants using the ground projection of the foot markers. No differences or trends were found between groups for any of the metrics (p-values of non-parametric Wilcoxon rank-sum tests are displayed), indicating there was no systematic difference in foot placement between both groups that could have affected the results.

1 SENSITIVITY ANALYSIS OF THE FPE

In the derivation of Eqn. 12 three assumptions have been made: that the inertia J of the model is constant, that the leg-length of the model is constant ℓ, and that the sum of kinetic and potential energy (after contact) is constant. To evaluate how much error these assumptions introduce into the calculations we begin by taking a first order series expansion of f in ϕ and J

$$f \approx f_0 + \frac{\partial f}{\partial \phi}\Delta\phi + \frac{\partial f}{\partial J}\Delta J. \tag{S1}$$

When ϕ satisfies the constraint $f = 0$ then $f_0 = 0$ and the equation can be rearranged to yield

$$\frac{\Delta\phi}{\Delta J} = -\frac{\partial f/\partial J}{\partial f/\partial \phi} \tag{S2}$$

which in the limit becomes

$$\frac{d\phi}{dJ} = -\frac{\partial f/\partial J}{\partial f/\partial \phi}. \tag{S3}$$

Similarly a first-order expansion of f in ϕ and ℓ about a value of ϕ which satisfies $f = 0$ yields

$$\frac{d\phi}{d\ell} = -\frac{\partial f/\partial \ell}{\partial f/\partial \phi}. \tag{S4}$$

Next we define system energy (E) as the sum of sum of kinetic (T) and potential energy (V)

$$E = T + V. \tag{S5}$$

To determine how the solution ϕ to $f = 0$ varies with changes in E we begin by noting that Eqn. 11 can be re-written as

$$\frac{1}{2}(J + m\ell^2)\omega_2^2 + mgh = mg\ell = E \tag{S6}$$

Using $mg\ell = E$, subtituting Eqn. 9 for ℓ, and taking a derivative we arrive at

$$\frac{d\phi}{dE} = \frac{\cos\phi}{mgh\tan\phi}. \tag{S7}$$

The rate at which the error in ϕ grows due to violations in these three assumptions is given by

$$\dot{\epsilon} = \frac{d\phi}{dJ}\dot{J} + \frac{d\phi}{d\ell}\dot{\ell} + \frac{d\phi}{dE}\dot{E} \tag{S8}$$

and the actual error expected at a specific time sample is given by

$$\epsilon = \dot{\epsilon}\Delta t \tag{S9}$$

where $\Delta t = 1/150$ is the time between samples. The symbolic expressions for the partial derivatives of f were formed using Maxima (2014) and evaluated numerically using the data from each experimental trial. The maximum values of the errors introduced by these assumptions in Table 1 show that the assumptions of constant J and ℓ introduce very little error. In contrast the assumption of a constant E is responsible for most of the observed error during seat-off when both the younger adults (120 [70.0]W) and older adults (58.0 [82.2]W) have values of $\dot{E}$ that are much larger than 0. However, because ϕ is updated at a rate of 150 Hz the error in ϕ for any single sample is at most 0.0258 rad (1.47°) for the younger adults and 0.0625 rad (3.58°) for the older adults.

	$\max\left(\frac{d\phi}{dJ}\dot{J}\right)$ rad	$\max\left(\frac{d\phi}{d\ell}\dot{\ell}\right)$ rad	$\max\left(\frac{d\phi}{dE}\dot{E}\right)$ rad	$\max(\dot{\epsilon})$ rad/s	$\max(\epsilon)$ rad
Y	0.00573	-0.00827	3.89	3.87	0.0258
O	0.00431	0.00209	9.37	9.37	0.0625

REFERENCES

Maxima. Maxima, a computer algebra system. version 5.34.1 (2014).

Table S1. Differences between younger and older adults at stance. The table compares the balance metric values at stance between the two groups. When comparing the group differences at stance to those at seat-off, most variables show considerably smaller differences at stance as expected (as can be seen in Fig. 5). Only larger differences found at stance are whole body angular speed and FPE -COP in $\hat{t}$, of which only angular speed is significantly different at seat-off. Both metrics have small group differences at seat-off and at stance, resulting in large relative differences that are small in practical terms. Overall, the trajectories of younger and older adults seem to converge from seat-off to stance, with small differences at stance.

**Parameter**	**Rank Sum** p-values	Differences between group (O-Y) Stance vs. Seat-off (%)
COM_{GP} to edge BOS	0.24	16.2
COM_{GP} to COP	0.57	1.6
COM speed	0.27	21.3
Angular speed	**0.04**	**119.6**
Angular velocity about the vertical	0.63	32.7
FPE to edge BOS	0.12	47.2
FPE -COP in $\hat{t}$	0.15	**407.9**
FPE -COP in $\hat{s}$	0.36	10.2

Table S2. Differences in within-subject variability between younger and older adults at seat-off. The table compares the within-subject variability, taken as the range of values per individual, of the duration and balance metric values at seat-off between the two groups. No difference was found between the variability displayed in younger versus older adults.

**Parameter**	**Rank Sum** p-values
total duration	0.348
duration seat-off to stance	0.118
COM_{GP} to edge BOS	0.949
COM_{GP} to COP	0.586
COM speed	0.586
Angular speed	0.286
Angular velocity about the vertical	0.743
FPE to edge BOS	0.973
FPE -COP in $\hat{t}$	0.381
FPE -COP in $\hat{s}$	0.448

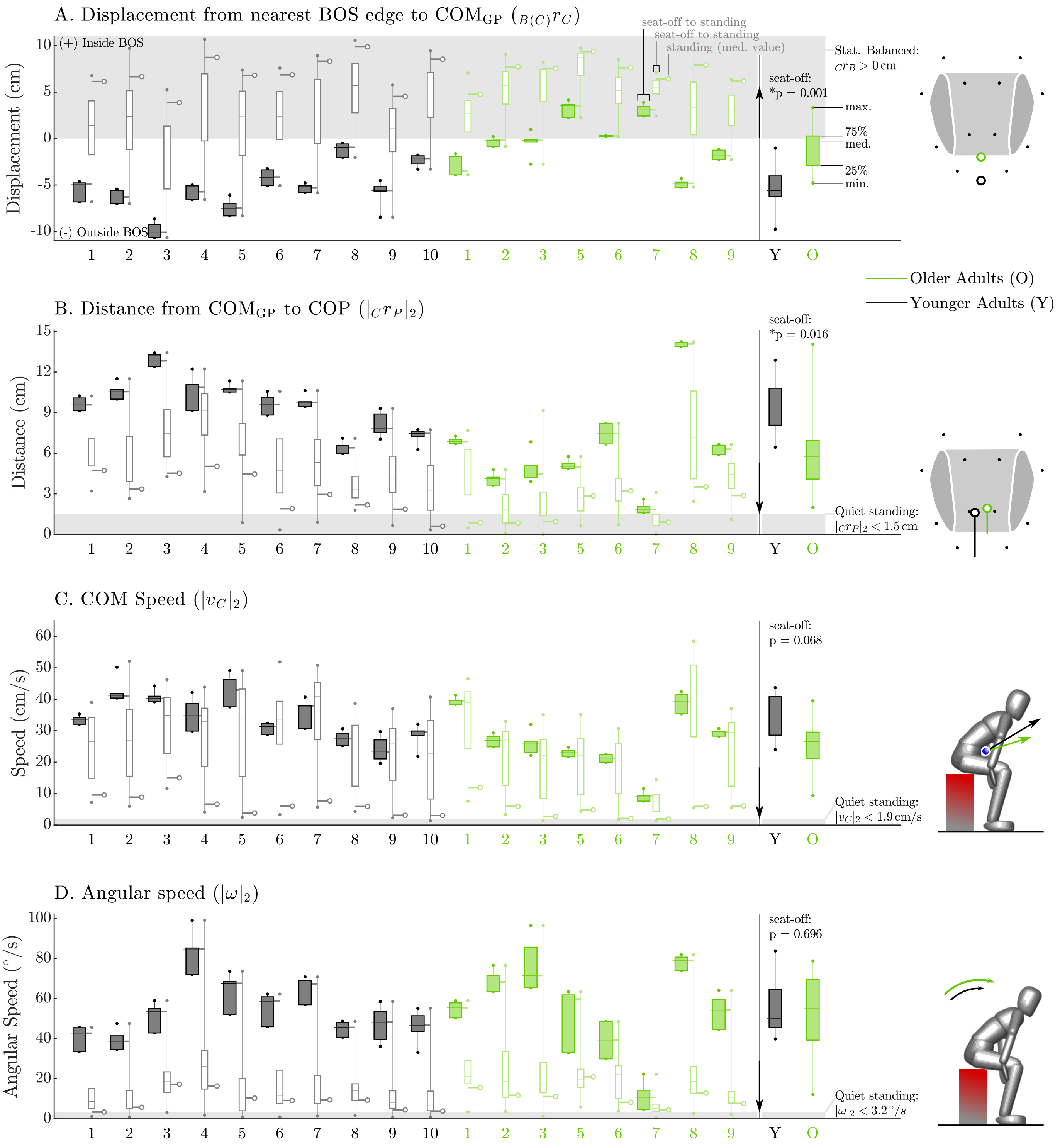

Figure S2. Static balance metrics for the Chest condition. Similar to the Side condition the older participants (green) position their COM$_{GP}$ more conservatively within the BOS **(A)**, and the COP closer to the COM$_{GP}$ **(B)**, than the younger participants (black). The speed of the COM in older adults is slower than the younger adults **(C)**, though the difference does not reach levels of significance. As with the Side condition the angular speed of both the older and younger participants is quite high at seat-off **(D)**. The static balance region is indicated with a grey box in each plot. The arrow between the individual and group data indicates the direction towards more conservative static balance.

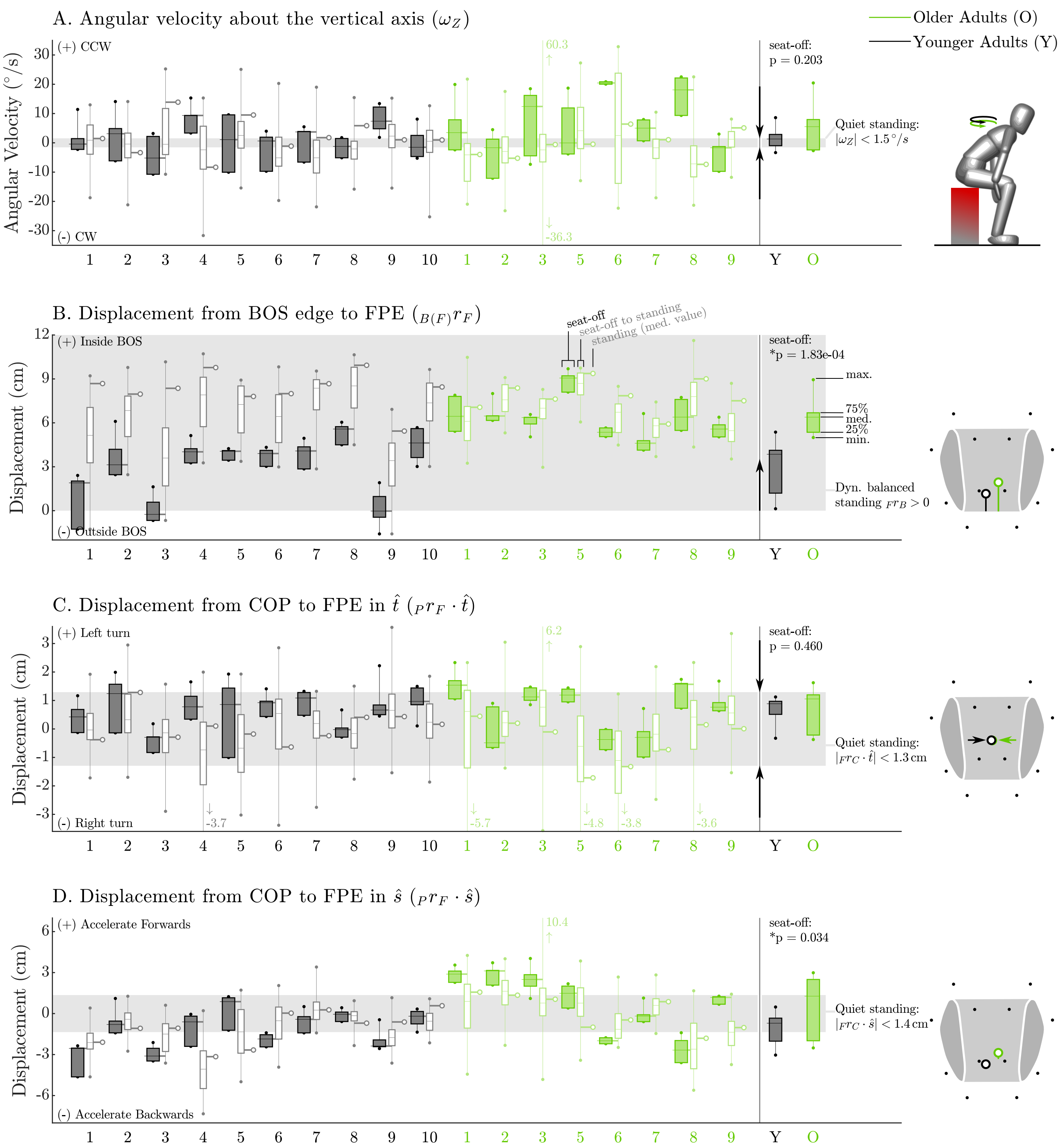

Figure S3. Dynamic balance metrics for the Chest condition. The assumption of the FPE that $\omega_{avg} \cdot \hat{k}$ is small is well met by most participants **(A)**. The variation in O6's values are dramatically smaller than the Side condition, likely because the arms are fixed. Both groups keep the FPE within the BOS **(B)** but Y1, Y3, and Y9 have some trials in which the FPE is outside of the BOS likely because the BOS model does not fit these participants accurately. As with the Side condition both groups display a tight control of the COP relative to the FPE location in the turning direction $\hat{t}$ **(C)**. The differences between the COP to FPE location are smaller in $\hat{s}$ **(D)** compared to the Side condition likely because the arms are not free to move. The arrows between the individual and group data indicate the direction towards a state of more conservative dynamic balance. No arrow appears in the plot of ${}_{\mathrm{P}}r_{\mathrm{F}} \cdot \hat{s}$ **(D)** as deviations in $\hat{s}$ are permitted (and thus indicate individual preference) provided ${}_{\mathrm{B(F)}}d_{\mathrm{F}}$ is positive.
